# Supplementary material for: Worker Size Diversity Has No Effect on Overwintering Success under Natural Conditions in the Ant Temnothorax nylanderi
Source: Insects. 2021 Apr 22;12(5):379. doi: 10.3390/insects12050379 (PMC8143561; doi:10.3390/insects12050379)
Supplement: Supplementary file 1 [file insects-12-00379-s001.zip › insects-1181864-supplementary.pdf]

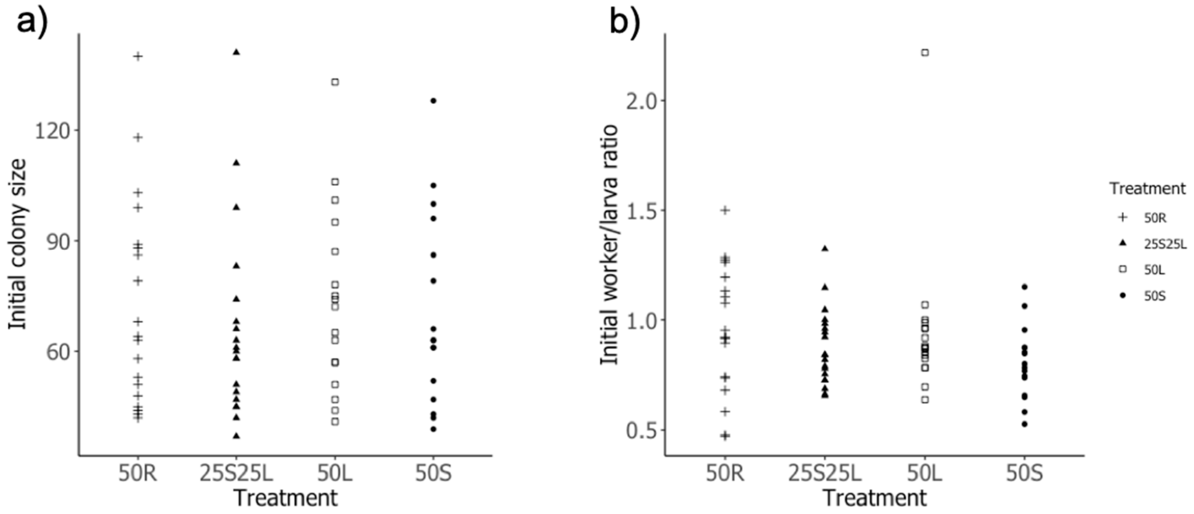


**Figure S1.** Plots comparing the distribution of (**a**) the initial colony size and (**b**) the initial worker to larva ratio depending on treatment (50R: random removal of 50% of workers; 25S25L: removal of the 25% smallest and the 25% largest workers; 50L: removal of the 50% largest workers; 50S: removal of the 50% smallest workers.). Twenty colonies were assigned to each treatment at the beginning of the experiment.

**Table S1.** The statistical power based on the observed biological effect size for each comparison for each variable. The biological effect size was estimated as Cohen's $d = (mean_2 - mean_1) / SD_{pooled}$ [87].

| Variables | Treatment comparison | Calculated effect size | Calculated power |
|---|---|---|---|
| Rate of workers gain/loss | 50L-50S | 0.39 | 0.82 |
| | 50L-50R | 0.29 | 0.55 |
| | 50L-25S25L | 0.07 | 0.07 |
| | 50R-25S25L | 0.25 | 0.42 |
| | 50S-25S25L | 0.21 | 0.30 |
| | 50S-50R | 0.56 | 0.99 |
| Rate of larvae gain/loss | 50L-50S | 0.21 | 0.30 |
| | 50L-50R | 0.12 | 0.12 |
| | 50L-25S25L | 0.20 | 0.28 |
| | 50R-25S25L | 0.15 | 0.17 |
| | 50S-25S25L | 0.03 | 0.05 |
| | 50S-50R | 0.13 | 0.14 |
